# suicide among women, its clinical specificities and risk factors

**DOI:** 10.1192/j.eurpsy.2023.2362

**Published:** 2023-07-19

**Authors:** K. Douk, I. belabess, H. Nafiaa, A. Ouanass

**Affiliations:** 1 psychiatry, Military hospital Mohammed V, Rabat; 2 psychiatry, psychiatric hospital ar-razi, salé, Morocco

## Abstract

**Introduction:**

In the general population, the authors have noted that most suicides are commited by men, this gender difference also includes the methods that suicidal people use to commit suicide; women tend to use less immediately lethal means such as drug ingestion in contrast to the methods that men adopt, which often include poisoning, hanging or the use of firearms. This is partly due to the fact that men verbalize their suffering less in the context of the hegemony of masculinity which delays the request for help.

**Objectives:**

Our objective is to describe the profile of female patients admitted to the AR-RAZI hospital in Salé for suicide attempts and/or with a previous suicide attempts and their treatment and to identify the risk factors, which will allow us to develop preventive therapeutic strategies, taking into account their age, their reasons for hospitalization and clinical presentation on admission; their physiological characteristics, their somatic and psychiatric co-morbidities and their socio-cultural factors.

**Methods:**

We have performed a cross-sectional study of 59 female patients admitted to Ar-Razi Psychiatric Hospital for suicide attempt or having already committed at least one suicide attempt, by means of a questionnaire specifying their age, marital status, occupation, socioeconomic level and residence.

The patients in question were recruited within a 2-year time frame, coming for the most part from the regions of the kingdom that our center covers.

**Results:**

- 13 are between 21-25 years old, 10 are between 15-20 years old, 9 are over 51 years old, 7 are between 31-35, 6 are between 26-30, 4 are between 41-45 and only one patient who is in the age range between 46-50.
- 31 are single, 16 are married, 11 are divorced or in the process of being divorced and only one widow.
- 36 have children between 1 and 6 years old and 23 have never given birth.
- 39 women never had a job or have not had one for at least 2 years, 10 are students, 5 are working in the public sector, 3 are working in the private sector and 2 are living from personal or family businesses.
- 30 live in a modest socioeconomic level, 25 in a medium level and 4 in a high level.
- 21 patients live in Rabat, 14 in Salé, 4 in Témara, and the rest are spread over the urban, rural and urban periphery of the territory that our center covers.

**Conclusions:**

The notion of suicide is quite wide to be reduced to suicide attempts, given the multitude of semiological equivalents of the suicidal act. apart from the etiology, the sex of the suicidal person strongly influences all the parameters of the suicidal person and the suicidal act, including the risk factors, the means used and the means of protection, hence the interest in adapting the preventive, diagnostic and therapeutic approach, both medical and social, by taking into account the sex of the suicidal person for optimal care.

**Disclosure of Interest:**

None Declared

